# Human Embryonic Kidney HEK293 Cells as a Model to Study SMVT-Independent Transport of Biotin and Biotin-Furnished Nanoparticles in Targeted Therapy

**DOI:** 10.3390/ijms26041594

**Published:** 2025-02-13

**Authors:** Magdalena Twardowska, Andrzej Łyskowski, Maria Misiorek, Żaneta Szymaszek, Stanisław Wołowiec, Magdalena Dąbrowska, Łukasz Uram

**Affiliations:** 1 The Faculty of Chemistry, Rzeszow University of Technology, Powstancow Warszawy 6 Ave., 35-959 Rzeszow, Poland; mczygier@prz.edu.pl (M.M.); zaneta.szymaszek212@gmail.com (Ż.S.); luram@prz.edu.pl (Ł.U.); 2 Medical College, University of Rzeszow, 1a Warzywna Street, 35-310 Rzeszow, Poland; swolowiec@ur.edu.pl; 3 Laboratory of Molecular Bases of Ageing, Nencki Institute of Experimental Biology, Polish Academy of Sciences, 3 Pasteur Street, 02-093 Warszawa, Poland; m.dabrowska@nencki.edu.pl

**Keywords:** PAMAM dendrimers, human embryonic kidney HEK293, biotin conjugates, neutral and cationic nanoparticles, SMVT-mediated transport, MCT-1, cellular uptake, targeted therapy

## Abstract

The aim of this study was to investigate the usefulness of human embryonic kidney HEK293 cells as a model of normal cells in biotin-mediated therapy. The expression and role of sodium multivitamin transporter (SMVT) in the uptake and accumulation of free biotin, as well as cationic and neutral biotinylated PAMAM dendrimers of the fourth generation synthesized in our laboratory, were assessed in HEK293 cells in comparison to other immortalized (HaCaT) and cancer cells (HepG2, U-118 MG). The obtained data showed that a higher level of SMVT in HEK293 cells was not associated with a stronger uptake of biotin and biotinylated PAMAM dendrimers. Biotinylation increased the selective uptake of neutral dendrimers in an inversely proportional manner to the concentration used; however, the accumulation in HEK293 cells was lower than that in cells of other cell lines. The time-dependent biotin and biotinylated dendrimers uptake profiles differed significantly. Therefore, it should be assumed that the efficiency of biotinylated nanoparticles’ uptake depends on multiple cellular transport mechanisms. Toxicity tests showed significantly higher sensitivity to PAMAM conjugates for HEK293 cells than for HepG2 and HaCaT cells. Molecular modeling studies and the profile of biotin uptake suggest that not only SMVT but also monocarboxylate transporter 1 (MCT-1) may play an important role in the selective transport of biotin and biotinylated nanoparticles into cells. Due to the complexity of the problem, further studies are necessary. In summary, HEK293 cells can be considered a valuable model of normal cells in the study of biotin- targeted therapy using nanoparticles based on PAMAM dendrimers.

## 1. Introduction

Cancer is the leading cause of death worldwide, accounting for one in six deaths [1]. Every day, approximately 52,900 individuals are diagnosed, and more than 27,000 die from this disease [2]. The main methods of fighting cancer include surgery, radiation therapy, and chemotherapy as single treatments or in combination [3]. Targeted therapy focuses on delivering drugs to specific genes or proteins that are unique to cancer cells or the surrounding tissue that supports cancer growth. Its success depends on precise release of the drugs at the site of the disease, which helps to reduce unwanted side effects on healthy tissues [4]. To date, key cell surface receptors like CD71 [5], HER2 [6], TNFR [7], CD47 [8], EGFR [9], VEGFR, and MET [10] have been exploited as molecular targets in the recognition of cancer cells. However, these proteins are also expressed in many non-cancer cells, mainly epithelial cells [11,12,13], and keratinocytes [14,15]. This causes side effects in healthy tissues of the bone marrow, digestive system, and hair follicles [16].

Biotin has been shown to be potentially useful in targeted anticancer therapy. Biotin, also known as vitamin B7 or H, is an essential nutrient required for cell growth, proliferation, and differentiation. Rapidly proliferating tumor cells produce high levels of biotin receptors on their surfaces [17,18]. The *SLC5A6* gene, located on chromosome 2p23, encodes the sodium-dependent multivitamin transporter (SMVT), which facilitates the transport of biotin into cells [19]. The strategy of targeting SMVT not only enhances drug permeation by bypassing physiological, biochemical, and biological barriers but also facilitates precise delivery at the intracellular level. Hydrophilic drug molecules that cannot efficiently penetrate biological lipid membranes can be delivered using a transporter-targeted delivery system. It can therefore be concluded that biotinylation of anticancer agents has the potential to enhance their specificity in tumor targeting. SMVT is expressed in various tissues, including the placenta, intestines, brain, liver, lungs, kidneys, cornea, retina, and heart [20]. Notably, SMVT is often overexpressed in cancer cells compared to healthy cells [19]. Therefore, a comprehensive evaluation of SMVT expression under diverse physiological and pathological conditions is essential to elucidate its regulatory mechanisms [20]. In light of utility, vitamin B7 is considered to be a highly exploratory tumor-targeting small-molecular ligand capable of targeting SMVT [21].

Another mechanism of small-molecule transport across the cellular plasma membrane is that mediated by various isoforms of the monocarboxylate transporters (MCTs) [22]. Many tumors, including both solid and hematological malignancies, demonstrate increased MCT expression [23]. This highlights the potential of these transporters as therapeutic targets [24]. MCT-1, along with MCT-4 (encoded by the *SLC16A3* gene), has been extensively investigated for its role in facilitating the transport of monocarboxylates, such as lactate, pyruvate, and ketone bodies, across the cell membrane [25]. There is evidence that small molecules like biotin could be transported via MCT-1 [26,27]. The increasing understanding of MCT functions and their regulation has led to the development of novel targeted therapeutics, which have potential for clinical applications.

The development of biotinylated prodrugs, which can selectively accumulate in cancer cells but not in normal cells, holds significant promise for targeted delivery of chemotherapeutic agents [28]. However, biotin–drug conjugates face two significant challenges. First, only small amounts of the drug can be delivered, as only a single drug molecule can be attached to each biotin molecule; second, ligand–drug conjugates are very small, which results in their excretion by the kidney and potential reabsorption in the proximal tubules, leading to unwanted accumulation in the kidney. Therefore, polymeric carrier systems could serve as an alternative to avoid these limitations [29].

Polyamidoamine dendrimers (PAMAMs) are a versatile and reproducible type of nanocarriers that can be used for drug delivery. By adding specific ligands, PAMAMs can be engineered to target the receptors that are overexpressed on cancer cells’ surfaces [30]. PAMAMs are highly branched, nanoscale polymer structures with a three-dimensional design. Their size ranges from 1.1 nm in the 1.0 generation (1.0 G) to 9.8 nm in the 8.0 generation (8.0 G) [31]. Higher generations of PAMAMs (G4 and higher) are characterized by prolonged blood retention and reduced renal excretion [32,33]. Moreover, PAMAMs can be specifically tailored by changing their functional groups, allowing them to become either hydrophilic or hydrophobic. These modifications enhance a drug’s ability to adapt to changes in homeostasis, such as variations in pH and membrane potential [34]. Unfortunately, dendrimers have limited applications due to their toxicity [35,36]. Numerous approaches have been developed to modify the surfaces of PAMAM dendrimers and to adjust their characteristics. By replacing the cationic groups with neutral (-OH) or anionic functional groups (-COOH), it is possible to avoid electrostatic interactions with biological membranes, improving their biocompatibility for safer use in drug delivery [37]. The use of dendrimers is limited, as they do not qualify for Generally Recognized as Safe (GRAS) status. Hence, dendrimers have not yet achieved the same level of success in clinical settings as linear polymers, and many biomedical applications are still unexplored [38]. It is challenging to give a clear and straightforward answer regarding the biocompatibility of PAMAM dendrimers. This is because dendrimers come in various sizes, generations, molecular components, and surface charges, all of which can influence their permeability and toxicity [39]. Newly designed and synthesized modified dendrimers require comprehensive assessments of their suitability and safety.

There is an urgent need to clarify the influence of PAMAM dendrimers on various types of cells, including normal cells. Such knowledge can provide information on the side effects of dendrimers and help improve existing ones. Human embryonic kidney 293 (HEK293) cells are considered a standard model of normal human cells [40]. Nevertheless, the issue of their “normality” is still debated [41]. The HEK293 cell line was made immortal by transforming human embryonic kidney cells with a fragment of adenovirus type 5 DNA. This modification allows the cells to continuously divide and be used for extended research purposes [42]. Due to their ease of transfection, HEK293 cells have been widely used to produce proteins. They are also commonly employed in molecular screening, particularly within the pharmaceutical industry. Due to the kidney’s fragile filtration system and its critical role in filtering bodily fluids and eliminating waste, exposure to nanoparticles could potentially disrupt the structure and function of renal cells. To explore this possibility, researchers used the well-established human embryonic kidney (HEK293) cell line as a model system, as it is commonly employed to assess the cytotoxicity of various chemicals [43,44,45].

The goal of this study was to examine the utility of HEK293 cells as a model of non-cancer cells for testing the efficiency of the cellular transport and biocompatibility of biotinylated, cationic, or neutral nanoparticles associated with transport via the sodium multivitamin transporter (SMVT) or monocarboxylate transporter 1 (MCT-1). We hypothesized that the high level of SMVT expression in HEK293 cells may be responsible for enhanced uptake of biotin by these cells. As nanoparticle models, glycidylated and/or biotinylated fourth-generation PAMAM dendrimers were used. We examined how charge modifications influenced dendrimers’ uptake, accumulation, and cytotoxicity and whether they affected cell proliferation. Additionally, special attention was given to correlations between the expression of SMVT or MCT-1 and the method of transport utilized by the PAMAMs. Dendrimers are considered potential drug carriers in numerous (mostly anticancer) therapies. It is therefore beneficial to investigate their influence on diverse cellular populations to validate their safety and assess their possible application.

## 2. Results

### 2.1. SMVT Level

**Figure 1:**
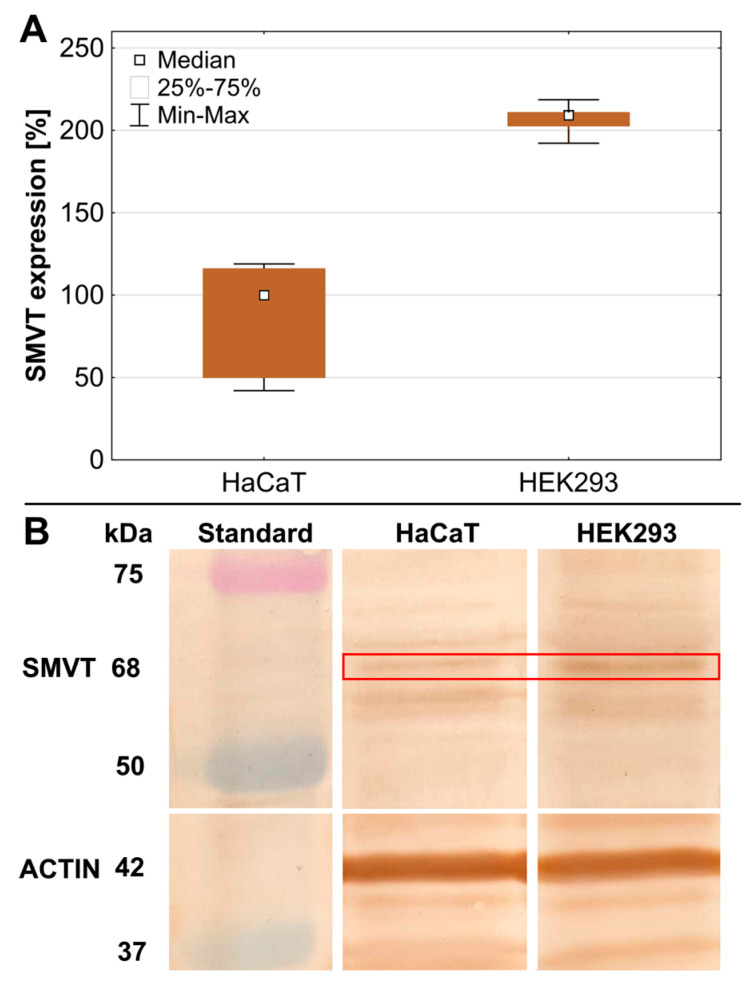
The expression of SMVT in HEK293 cells evaluated with the Western blot technique. (**A**) Levels of SMVT expression were quantified as percentages of the SMVT level in HaCaT cells. White squares indicate the medians; the lower (25%) and upper (75%) quartile ranges are presented as whiskers. (**B**) Obtained blots, where red box indicates SMVT bands (68 kDa).

To assess the level of SMVT expression in HEK293 cells, Western blot analysis was performed. The level of SMVT expression in the HEK293 cells was compared to that in human spontaneously immortalized keratinocytes (HaCaT) commonly used as a model of normal non-cancerous cells. The results showed a more than 2-fold higher SMVT level in HEK293 cells (209%) than in HaCaT cells (Figure 1).

Our earlier studies indicated that the expression of SMVT in HEK293 cells was also higher than that in U-118 MG glioma cells (134% of the HaCaT level) but lower than that in the case of hepatocellular carcinoma HepG2 cells (294% of the HaCaT level) [46]. The obtained results showed that the level of SMVT expression in HEK293 cells reached an intermediate value compared to that in different types of cancer cells (glioma and hepatocellular carcinoma cells).

### 2.2. Biotin Uptake Efficiency

**Figure 2:**
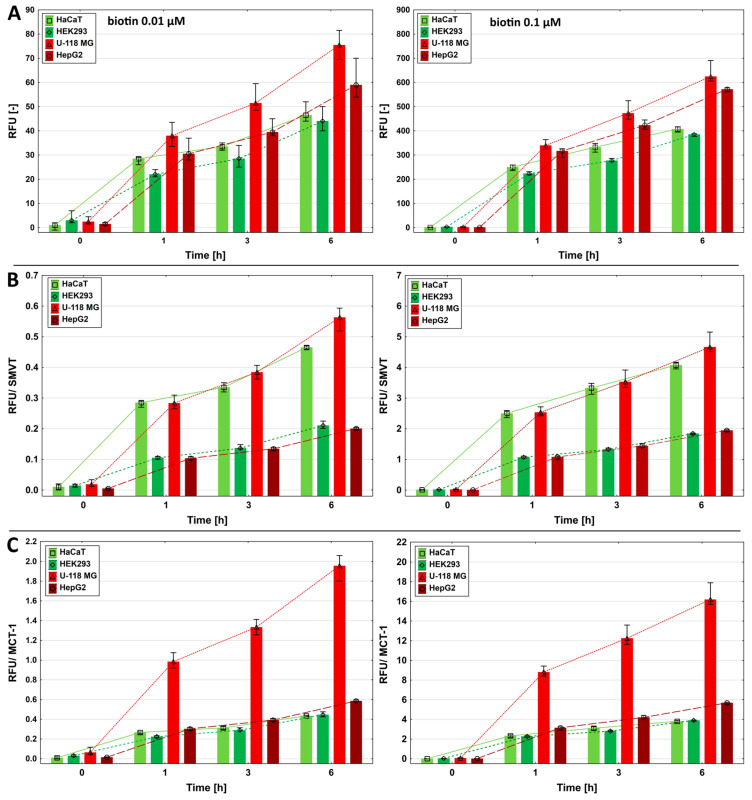
(**A**) The time- and dose-dependent uptake of fluorescently labeled biotin into HEK293 cells and comparatively into HaCaT, U-118 MG, and HepG2 cells, which were incubated at a 0.01 or 0.1 μM concentration for 1, 3, or 6 h. (**B**) Uptake in relation to the SMVT level determined by Western blot. (**C**) Uptake in relation to MCT-1 expression published in the Human Protein Atlas [45]. Results are presented as the medians, and whiskers indicate the first and third quartiles. Results for HaCaT, U-118 MG, and HepG2 cells were from [46].

Biotin fluorescently labeled with ATTO 590 was used to determine time- and concentration-dependent uptake. Uptake was measured after 1, 3, or 6 h of incubation at two ATTO 590-labeled biotin concentrations—0.1 or 0.01 μM. An increase in biotin uptake efficiency was observed with increasing incubation times and concentrations of this vitamin (Figure 2A).

The HEK293 cells, despite having more than twice the level of SMVT expression than HaCaT and U-118 MG cells, showed the lowest biotin uptake at each concentration and incubation time. For this reason, assuming that SMVT is the main biotin transporter, we estimated the efficiency of biotin transfer in relation to the SMVT level. Figure 2B shows that the transport efficiency of fluorescently labeled biotin by SMVT was significantly higher in U-118 MG and HaCaT cells than in HEK293 cells. HEK293 cells transferred biotin with a similar efficiency as HepG2 cells (Figure 2B). After calculating the biotin transport efficiency in relation to the MCT-1 level (data from the Human Protein Atlas [43]), an almost identical profile to that in the case of biotin transport alone was observed in HaCaT, HEK293, and HepG2 cells (Figure 2A,C). The exception was U-118 MG cells, which transported labeled biotin significantly more intensively.

### 2.3. Biotin and Derivatives’ Binding to the Monocarboxylate Transporter 1 (MCT-1)

**Figure 3:**
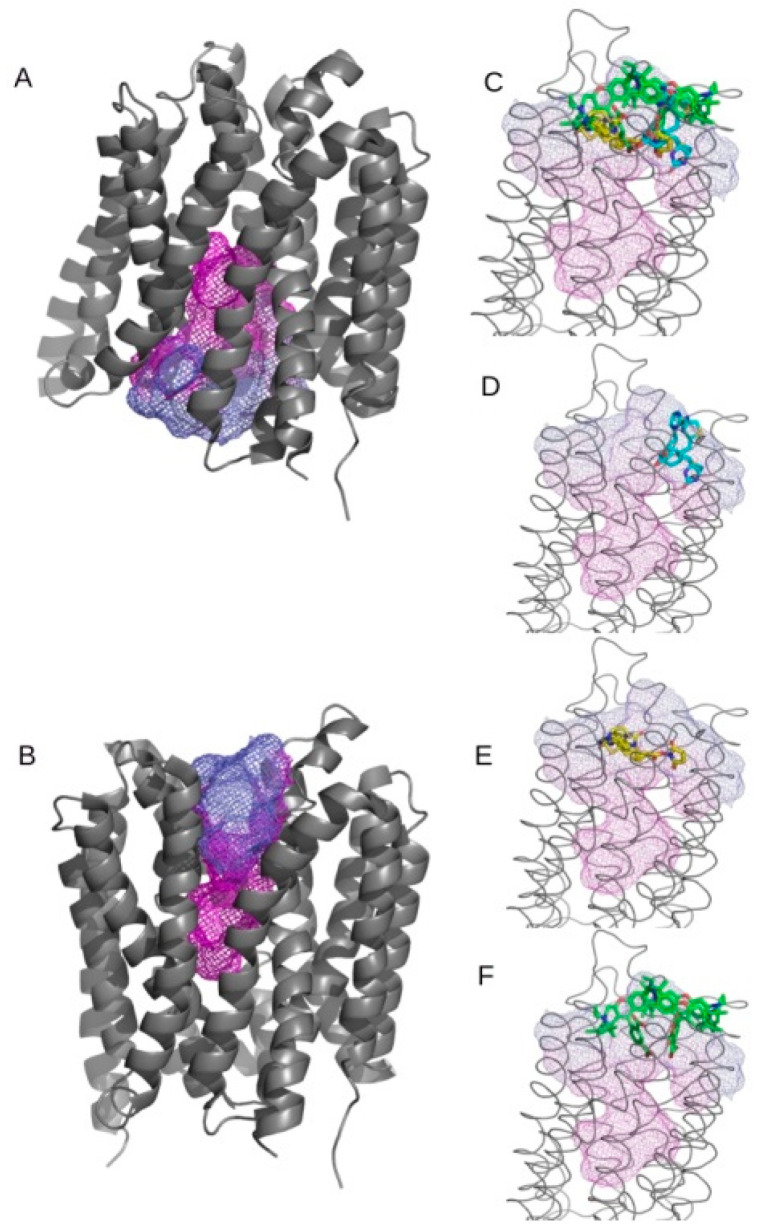
Panel (**A**) shows the overall structure of the MCT-1 transporter based on PDB ID: 7CKO representing an inward open configuration. Panel (**B**) depicts the overall structure of the MCT-1 transporter based on PDB ID: 6LYY representing an outward open configuration. Mesh represents cavities predicted by the CavitOmiX PyMOL plugin. Panels (**C**–**F**) present the results of the blind docking simulation. Ligands are presented as follows: biotin, light blue; biotin–NHS, yellow; and ATTO 590 fluorophore, green. Panels (**C**) presents clustered binding modes for all tested ligands. Panels (**D**–**F**) present the top three configurations for each tested ligand separately in the following order: biotin, biotin–NHS, and the ATTO 590 fluorophore. Results were visualized with the open-source build of PyMOL (open-source ver. 3.0.0).

In addition to SMVT, MCT-1 is considered to be the major biotin transporter. Therefore, we decided to test whether ATTO 590-labeled biotin could potentially bind to this transporter. A protein structure template search for a docking simulation revealed a number of experimental MCT-1 structures, which were mostly determined by electron microscopy as well as computational models generated by the AlphaFold project. The identified models represent two distinctive transporter conformations—outward and inward open. When additionally available AlphaFold computational models (AlphaFold ID: AF-A0A024R0H1-F1-v4, AF-P53985-F1-v4) were aligned with the respective experimental structures, the RMSD was much better for the AlphaFold inward open conformation at 0.836 Å and 0.818 Å vs. 3.615 Å and 3.817 Å, respectively, for the computational models and structures PDB ID: 7CKO and PDB ID: 6LLY [47] (Figure 3A,B).

The structure 6LLY, representing an outward open conformation, was selected for the docking simulation. The structure was downloaded directly from RCSB PDB database, and ligands present in the structure, as well as any additional polypeptides not relevant to the transporter, were manually removed. Cavity analysis revealed one major cavity localized centrally in the transporter core with a calculated volume of 2153 Å^3^. The remaining calculated cavities were much smaller, with values less than 215 Å^3^ each. The blind docking simulations were set with an outward open conformation and respective ligands. All tested ligands, including biotin, biotin–NHS, and the ATTO 590 fluorophore, displayed similar properties when tested with the same binding box center and size. All three ligands occupied a discrete space in the putative binding cavity. Biotin moieties occupied a similar space, while the ATTO 590 fluorophore localized nearby. The top three binding modes aligned well with the predicted cavity (Figure 3C). All configurations clustered near the central cavity core (Figure 3D–F).

In conclusion, the blind docking simulations revealed that the putative binding cavity is capable of binding all of the tested ligands. Furthermore, specific clustering of the top scoring configurations suggests that while the general localization of the binding site was shared among the tested molecules, each of them displayed a specific binding mode related to physicochemical properties. The distinctive binding pattern of ATTO 590 confirms that it is a viable probe for testing the transport properties of MCT-1.

### 2.4. Dendrimers’ Toxicity and Antiproliferative Action

**Figure 4:**
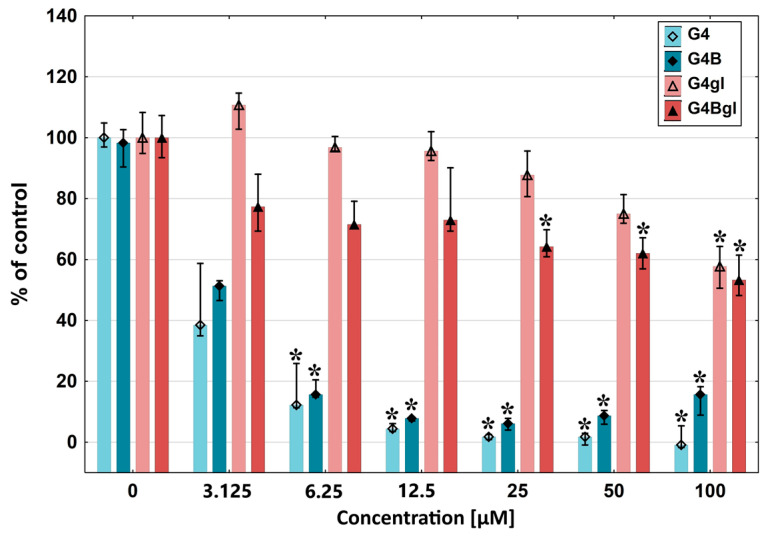
The G4, G4B, G4gl, and G4Bgl dendrimers’ cytotoxicity against HEK293 cells assessed after 48 h of incubation using the XTT assay. Cell viability is expressed as the median percentage against that of a non-treated control (control expressed as 100%). The whiskers are the lower (25%) and upper (75%) quartile ranges. * *p* ≤ 0.05; Kruskal–Wallis test (against the non-treated control).

The influence of biotinylation on the cellular uptake of PAMAM dendrimers was examined. Establishing a toxicity profile was a primary step to evaluate the biocompatibility of the tested compounds. An assay with tetrazolium salts (XTT) performed after 48 h of incubation with the studied dendrimers showed that the native PAMAM G4 was the most toxic, which caused a decrease in cell viability up to 40% in a concentration range of 3.125–100 μM. Biotinylated PAMAM G4 (G4B) was slightly less aggressive but still had a significant impact. The cell viability difference between these two compounds was highest at 3.125 μM and 100 μM concentrations, with values of 13% and 15%, respectively. At higher concentrations, significant differences were not seen. Glycidylated PAMAM G4 (G4gl) and both biotinylated and glycidylated PAMAM (G4Bgl) turned out to be less toxic. G4gl was the safest for cells. The statistically significant decrease in HEK293 cell viability induced by G4gl was observed only at the 100 μM concentration (cell viability equal to 58%), whereas the biotinylated analog G4Bgl induced toxicity starting from the 25 μM concentration, with cell viability equal to 62% (Figure 4).

**Figure 5:**
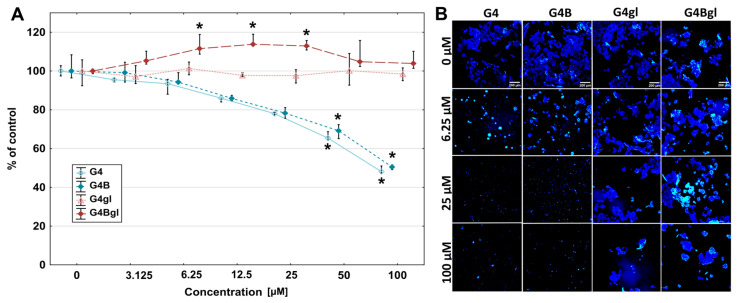
(**A**) The effects of the G4, G4B, G4gl, and G4Bgl dendrimers on HEK293 cell proliferation after 48 h of incubation. Cell viability is expressed as the median percentage against that of the non-treated control (control expressed as 100%). The whiskers are the lower (25%) and upper (75%) quartile ranges. * *p* ≤ 0.05; Kruskal–Wallis test (against the non-treated control). (**B**) Images of HEK293 cell nuclei from a fluorescence microscope after labeling with DAPI (blue signal).

In order to assess the degree of proliferation, an assay to determine the amount of DNA was performed. The results showed an antiproliferative effect of G4 and G4B, which was consistent with the decreased cellular viability observed in the XTT assay. At the highest concentrations of 50 and 100 μM, G4 and G4B caused reductions in cell proliferation by approximately 30 and 50%, respectively. Glycidylated dendrimer (G4gl) had no significant influence on cell division across the whole range of tested concentrations. Glycidylated and biotinylated PAMAM G4 (G4Bgl) increased cell proliferation. In the 6.25–25 μM concentration range, cell proliferation was higher by approximately 11–15%, and these differences were significant compared to the results for the control (no dendrimer treatment) (Figure 5A).

The obtained data were confirmed by fluorescence microscopy images, showing a different number of cells depending on the dendrimer used. G4 and G4B inhibited cell division from the lowest concentrations used, while glycidylated and/or biotinylated dendrimers showed inhibition only at the highest concentrations. Additionally, an increase in cell density was noted for G4Bgl-treated cells in the concentration range of 6.25–25 µM (Figure 5B).

### 2.5. Time- and Dose-Dependent Dendrimer Uptake

**Figure 6:**
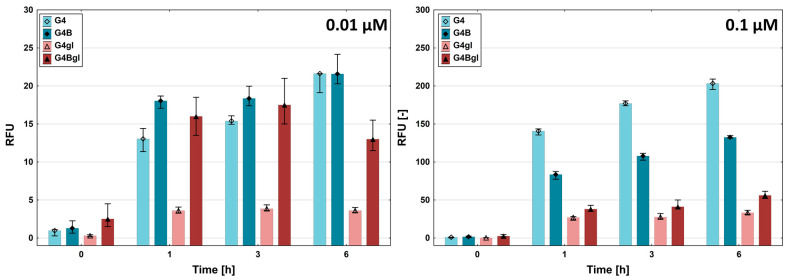
The time-dependent uptake efficiency of the Cy-5-labeled PAMAM dendrimers (G4, G4B, G4gl, and G4Bgl) by HEK293 cells. The cells were incubated for 1, 3, or 6 h with dendrimer concentrations of 0.01 or 0.1 µM.

To evaluate the efficiency of PAMAM dendrimer conjugates’ uptake, a time-dependent assay was performed. After 1, 3, or 6 h of incubation with fluorescently labeled dendrimers at two concentrations (0.01 and 0.1 μM), fluorescence was measured. The effects depended on the dendrimer concentration. Biotinylated G4 PAMAMs (G4B and G4Bgl) penetrated HEK293 cells at a higher level than native and glycidylated PAMAMs (G4, G4gl) during the initial three hours of incubation at the lower 0.01 μM concentration. Furthermore, the maximal level of G4B was constant up to the end of incubation. Similarly, -OH-terminated PAMAM G4 (G4gl) exhibited a comparable level during the entire incubation time. Following a six-hour incubation period, the uptake of native G4 was at the same level as that of G4B (Figure 6).

However, at the 0.1 μM dendrimer concentration, the effect was totally different. Cationic G4 exhibited the greatest uptake level during the entire experiment, which was proportional to the incubation time. G4B produced similar results but showed significantly lower uptake than G4 at all time points. Neutral PAMAMs (terminated with glycidol) showed significantly lower uptake than cationic PAMAMs, and their levels at each time point were rather constant. However, the amount of biotinylated and glycidylated G4Bgl uptake was higher than that of G4gl uptake. Thus, biotinylation apparently increased the penetration of neutral nanoparticles into HEK293 cells.

### 2.6. Dendrimer Accumulation

**Figure 7:**
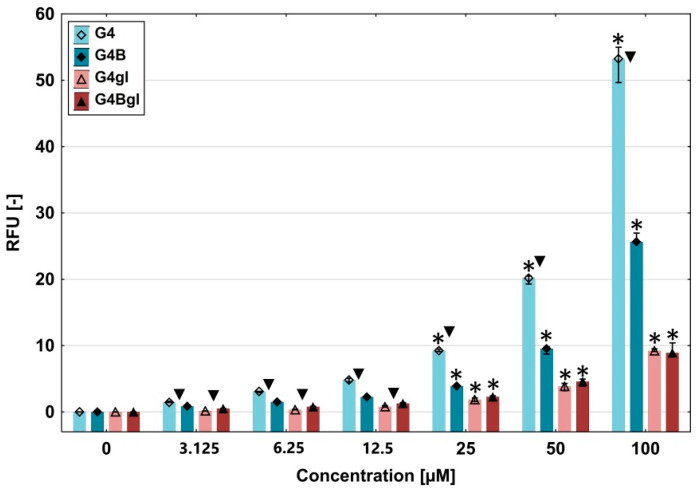
The accumulation of G4, G4B, G4gl, and G4Bgl dendrimers in HEK293 cells (per cell) after 48 h of incubation at a concentration range of 3.125 to 100 μM. Results are presented as the median, and whiskers indicate the first and third quartiles. * *p* ≤ 0.05; Kruskal–Wallis test (against the non-treated control). ▼ ≤ 0.05; Mann–Whitney U test.

In order to determine the ability of the fluorescently labeled dendrimers to accumulate in HEK293 cells, a retention assay was performed after 48 h of incubation. The unmodified G4 PAMAM dendrimer accumulated the most in HEK293 cells, and the increase in accumulation was directly proportional to the compound concentration. A similar pattern was observed for biotinylated dendrimers, but biotin attachment resulted in a diminished bioconjugate amount in cells. The attachment of glycidol to dendrimer conjugates caused significant decreases in their levels in cells. At the lowest 3.125 µM concentration, the accumulation of all dendrimers in HEK293 cells was hardly noticeable. At the highest concentrations of 50 and 100 µM, the attachment of biotin to the G4 PAMAM resulted in an approximately 2-fold decrease in the accumulation level, while biotinylation of G4gl did not result in higher penetration into the cells. The levels of these dendrimers (G4gl, G4Bgl) were comparable for all concentrations applied (Figure 7).

**Figure 8:**
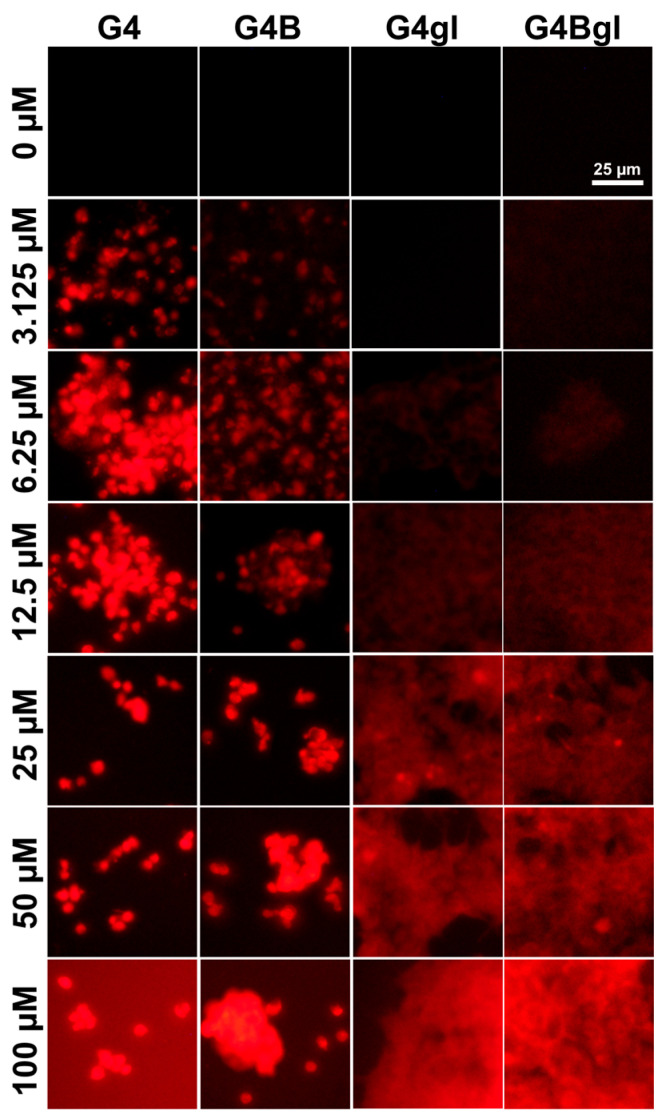
Fluorescence microscopy images presenting the amount of fluorescently labeled G4, G4B, G4gl, and G4Bgl PAMAM dendrimers (red signal) in live HEK293 cells after 48 h of incubation at concentrations ranging from 3.125 to 100 μM. The images do not reflect the same number of cells because G4 and G4B showed high cytotoxicity against HEK293 cells.

The results presented in Figure 7 are in agreement with the images obtained by fluorescence microscopy (Figure 8).

In the case of the native and biotinylated dendrimers, the number of live, adherent cells was reduced, confirming a significant influence of these dendrimers on HEK293 cell viability (Figure 5). Additionally, cells grew in separate clusters. G4 and G4B penetrated into cells and accumulated in the cytoplasm and nuclei. Glycidylated PAMAMs had no effect on cell morphology, which is probably related to their higher biocompatibility (Figure 4 and Figure 5). The increase in proliferation levels after G4Bgl incubation, as detected by quantitative measurement with DAPI dye (6.25–25 µM, Figure 5), was also evident from the higher amount of accumulated dendrimer, which is indicated by red fluorescence in Figure 8. Glycidylated dendrimers entered cells and accumulated in the cytoplasm, highlighting the cell shape. Spots of stronger fluorescence can be seen in the images, representing nuclei and other compartments (e.g., vesicles and lysosomes).

## 3. Discussion

Biotin is transported into cells mainly by SMVT. This transporter is responsible for the uptake of several essential vitamins, including biotin, pantothenic acid (vitamin B5), and lipoic acid [48]. The mechanisms of biotin transport into cells have been studied for years, but they have not been exactly delineated to date. Many cell lines characterized by specific expression of biotin transporters (SMVT, MCT-1, and FATP1—fatty acid transport protein 1) and many types of biotinylated drugs and nanoparticles were included in these studies. The HEK293 cell line is frequently used as an in vitro research model, including in the field of biotin transport research [49,50,51]. However, the level of naturally occurring SMVT in HEK293 cells during in vitro culture has never been determined. These cells are not exactly considered normal, but they are referred to as immortal, non-carcinogenic, and embryonic. Since SMVT is viewed as the main factor responsible for biotin transport, we determined its level in HEK293 cells. We proved that SMVT expression in HEK293 cells was higher than that in HaCaT and U-118 MG cells but lower than that in HepG2 cells. The obtained data are in agreement with the Human Protein Atlas [52], with the exception of U-118 MG cells. In the next step, we checked whether the rate of biotin transport correlated with the level of SMVT. Analysis of the uptake rate of biotin normalized to the expression of SMVT showed that in cells of the cell lines tested, SMVT transported biotin with varying efficiency. In HEK293 and HepG2 cells, SMVT-dependent biotin transport was 2.5-fold lower than that in U-118 MG and HaCaT cells. This finding indicates that SMVT may not be the only cellular biotin transporter. In the literature, it is often stated that biotin should be a factor in therapeutic agents, especially anticancer drugs, targeting cells that overexpress SMVT [53]. It was shown that some types of cancer cells indeed overexpress SMVT [20,54]. Nonetheless, other factors responsible for biotin transport should be considered. In HaCaT cells, MCT-1 was suggested to be responsible for more efficient biotin transport than that observed in HepG2 cells, which showed higher SMVT expression levels [55]. Also, in HEK293 cells, MCT-1 expression was confirmed [54,56]. In our previous studies, we showed that biotin and ATTO 590-labeled biotin demonstrate an affinity for similar putative binding pockets of SMVT that are fragments of a larger binding surface able to accommodate the fluorophore of ATTO 590 [46]. Meanwhile, Tripathi et al. showed that, based on a comparison of the results of many studies, SMVT can only transport compounds that have a free carboxyl group [57]. In our studies, biotin had a carboxyl group blocked by the presence of the ATTO 590 fluorophore, which, according to Tripathi et al., prevented its transport by this transporter. Therefore, we tested the possibility of MCT-1 as a transporter of biotin and ATTO 590-labeled biotin. We showed by molecular modeling that the MCT-1 molecule was capable of biotin binding (Figure 3). Thus, the involvement of MCT-1 in the cellular uptake of biotin should be considered.

The phenomenon of biotin uptake by cells has been extensively studied by other groups. Saha et al. tested it in HEK293 cells incubated with 10 nM biotin. They concluded that biotin entered cells not only by SMVT but also by passive diffusion [49]. This appears unfeasible in case of PAMAP conjugates due to the considerable sizes of the molecules. Biotin is a relatively small molecule, with a molecular size of 244 Da [58], but the mass of fluorescence (ATTO 590)-labeled biotin is around 1 kDa. It is known that the cell membrane is permeable to molecules smaller than 1 kDa [59]. Being at the permeability limit indicates that it is therefore unlikely that biotin–ATTO 590 could be taken up by cells via passive diffusion. However, other studies report that biotin uptake across cell membranes may occur through passive diffusion when extracellular biotin levels are above 25 μM, but at concentrations below 5 μM, uptake is primarily driven by carrier-mediated mechanisms [60]. Even if we assume that biotin–ATTO 590 could enter via passive diffusion, the concentrations that we tested were much lower than those favoring passive diffusion. Our current data can be compared to the results of our previous studies [46], where HaCaT cells were shown to take up biotin at a slightly higher rate than HEK293 cells despite SMVT being expressed at a 2-fold lower level in these cells than in HEK293 cells. This raises the question of whether the presence of SMVT protein directly correlates with the level of biotin uptake. This issue was also studied by others in T47D MCF-12A cells, and it was concluded that a higher level of SMVT resulted in higher internalization of biotin [61]. However, considering our results, it is not clear whether the cell lines selected were appropriate models for biotin and biotinylated product uptake that can be compared to the cell lines employed in our studies. It is currently believed that SMVT is the primary transporter for biotin internalization [48]. Nevertheless, some researchers postulate that transport can also be carried out by MCT family transporters [57,62]. Our results showed that after normalizing biotin transport efficiency to MCT-1 expression, nearly identical profiles of biotin absorption were observed for HaCaT, HEK293, and HepG2 cells. The exception was glioma U-118 MG cells, which transported biotin significantly more intensively. This suggests that MCT-1 is more likely to be a transporter of biotin than SMVT. At the same time, U-118 MG cells may have another mechanism in addition to SMVT and MCT-1 to increase the uptake of this vitamin. It is evident from our current studies that in HEK293 cells, the transport of biotin was not proportional to the level of SMVT protein. Similar results were obtained by Vadlapudi et al. where the uptake of biotin by HCEC cells (with a lower amount of SMVT) was higher than that by D407 cells (with a higher amount of SMVT) [63].

Dendrimers have significantly advanced the medical field as potential nanocarriers. However, cytotoxicity remains the main obstacle to their safe application. Due to the nanoscale dimensions, dendrimers can interact with various cellular components, such as the plasma membrane, organelles (e.g., mitochondria and nuclei), proteins, heavy metals, ions, vitamins, and nucleic acids [64]. It is well known that the cytotoxicity of dendrimers is strongly influenced by the quantity and type of functional groups on their surfaces. Cationic dendrimers tend to exhibit significant toxicity, while anionic and neutral dendrimers generally display minimal or no toxic effects [65]. In our current study, the influence of biotinylation on the uptake and accumulation of PAMAM dendrimers, including cationic (native G4) and neutral (glycidol-flanked G4gl) dendrimers, in HEK293 cells was investigated. The XTT assay showed that both native dendrimers, G4 and G4B, were the most toxic, which is most likely attributed to their cationic character that leads to disruption of cell membrane continuity. PAMAM G4 was the most toxic, probably due to -NH_2_-terminating groups on the surface. A comparable effect was observed in the case of G4B, which also possesses many non-blocked amine groups. Similar results were demonstrated by Srinageshwar et al. [66]. Glycidol-terminated dendrimer G4 was significantly more biocompatible for HEK293 cells. In many studies, including ours, neutral -OH-terminated PAMAMs exhibited less toxic effects than cationic ones [46,67,68]. Biotinylated G4gl displayed intermediate but still very low toxicity and lower biocompatibility than native G4gl. When applied at high concentrations, G4Bgl showed much higher biocompatibility than G4 and G4B. The slightly higher toxicity of biotinylated G4Bgl than that of G4gl was probably due to the greater uptake of these nanoparticles caused by the presence of biotin. Other studies also highlighted biotin as a molecule that can increase the toxicity of nanoparticles [69,70,71]. However, biotinylation of the cationic dendrimer did not significantly increase its absorption (especially at the higher 0.1 µM concentration), and it had no effect on toxicity. The similar or slightly weaker effect of biotinylated G4B than native G4 could result from the protective properties of biotin itself, which is known to be an essential nutrient required for cell growth, proliferation, and differentiation. It was shown that during in vitro culture of oligodendrocytes, biotin protected cells from metabolic injury, enhanced myelin-like ensheathment, and increased ATP production [72].

In addition, a cell proliferation assay was carried out in order to verify the dendrimers’ mechanism of cytotoxicity. The decrease in HEK293 cell number through treatment with cationic G4 and G4B was probably the result of the toxic effect on mitochondria, which occurred at the two highest concentrations (50 and 100 µM). It was proven that second- and third-generation PAMAM dendrimers caused mitochondrial damage and abnormalities in cell differentiation [73]. The neutral G4gl and G4Bgl dendrimers did not disturb HEK293 cell proliferation. Moreover, biotin contained in the G4Bgl conjugate was also found to stimulate cell proliferation in a concentration range of 6.25–25 µM. This is consistent with biotin being an indispensable nutrient for rapidly growing HEK293 cells [74]. In light of our previous studies, it can be concluded that HEK293 cells’ tolerance to the tested dendrimer conjugates was lower than that of HaCaT keratinocytes and liver cancer HepG2 cells but was similar to that of glioma U-118 MG cells.

Cationic PAMAM dendrimers can be transported into cells via direct penetration or endocytosis pathways (clathrin-mediated pathways and macropinocytosis) [75]. A crucial condition for successful drug delivery by this mechanism is that the drug molecule can be cleaved from the conjugate within the endosome and enter the cytosol [76]. Cationic and neutral dendrimers are likely to be taken up through non-clathrin, non-caveolar pathways, potentially involving electrostatic interactions or other forms of non-specific fluid-phase endocytosis [77]. This explains the stronger permeation of the G4 dendrimer than the G4gl dendrimer observed in our studies. The biotinylated G4Bgl dendrimer was taken up approximately 3-fold more efficiently than the non-biotinylated analog. Biotinylated dendrimers (as other nanoparticles) do not have a precisely described mechanism of transport. Bruce et al. proved that biotin-conjugated PEG-poly(glutamic acid) (-OH-terminated as in the G4gl dendrimer) achieved intracellular delivery of the cargo in lung A549 epithelial cells in vitro via “biotin receptors”. They also claimed that these receptors were SMVT receptors but did not present evidence confirming this hypothesis. Moreover, this biotinylated and -OH-terminated complex entered A549 cells through a biotin receptor-mediated and caveola-dependent pathway, while its non-biotinylated analog entered through a dynamin-dependent clathrin pathway [78]. Tripathi et al. described that biotin conjugates may enter into cells through endocytosis, unknown pathways, or maybe via carrier-mediated transport (e.g., SMVT). The results from the competition assay of biotin conjugates with free biotin and endocytosis experiments imply the involvement of multiple mechanisms for the uptake of biotin conjugates [57]. Therefore, in our studies, we wanted to test whether the factor associated with more efficient uptake of biotinylated dendrimers (cationic and neutral) could be SMVT-related transport.

We assumed that if SMVT is responsible for the transport of biotinylated dendrimers, or if SMVT acts as an endocytosis receptor, the time- and concentration-dependent patterns of biotinylated dendrimers’ entry into cells would be similar to the uptake of biotin itself. The results showed that the time- and concentration-dependent efficiency of biotin entry into HEK293 cells differed significantly from the kinetics of biotinylated G4B and G4Bgl uptake, which excludes this possibility. Analogous conclusions can be drawn from the data concerning HaCaT, U-118 MG, and HepG2 cells, which are presented in our previous paper [46]. It is possible, however, that the uptake efficiency of biotinylated nanoparticles depends on different types of transport. The penetration of the native biotinylated G4B dendrimer into cells occurs via direct penetration or endocytosis pathways (clathrin-mediated pathways and macropinocytosis) [79], and at a low 0.01 µM concentration, it can be supported by biotin-related transport. In the case of glycidol-flanked dendrimers (–OH surface residues), biotin-related transport can be supported by a non-clathrin, non-caveola pathway, which is characteristic of neutrally charged nanoparticles.

The lower absorption efficiency of neutral dendrimers than cationic dendrimers is not a new observation. Similar results were obtained by Albertazzi et al., who indicated that cationic G6 PAMAM dendrimers accumulated at a higher level than neutral G6. Additionally, they demonstrated that cationic dendrimers exhibit a strong electrostatic attraction to negatively charged membrane proteoglycans and phospholipids, suggesting that their uptake occurs through an active energy-dependent process [79]. In our studies, -OH-terminated dendrimers (G4gl) exhibited the lowest rate of internalization. This can lead to the conclusion that biotin has potential as a targeting ligand that can facilitate transport into cells, but its utility should be considered in a specific concentration range because of the particular interactions between the surface moieties of nanoparticles and cells. The diameter of the nanoparticles under consideration will also play an important role.

**Figure 9:**
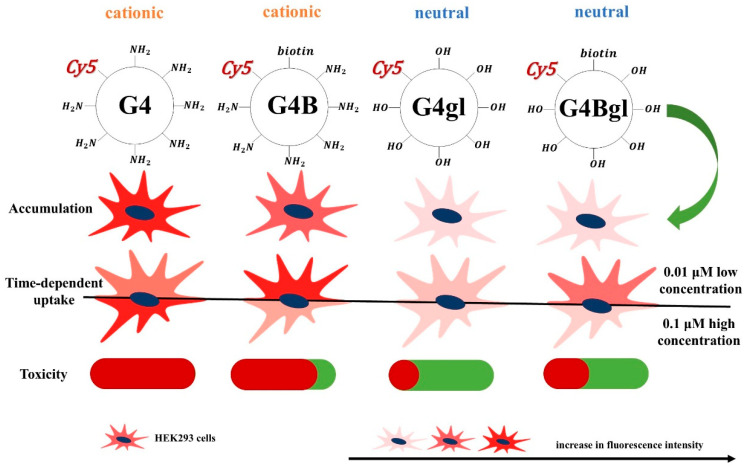
The relationships between the surface chemistry of G4, G4B, G4gl, and G4Bgl and cellular uptake, accumulation, and toxicity against HEK293 cells. In accumulation and time-dependent uptake rows the intensity of the red color indicates the intensity of fluorescence. In toxicity row red color indicates high toxicity and green color no toxicity.

Apart from the uptake process, the ability of nanoparticles to accumulate in cells for a long time and to avoid efflux is also an important issue. Our results show that all tested dendrimers accumulated in HEK293 cells. However, in relation to our previous studies, it is worth mentioning that the amount of the biotinylated G4Bgl dendrimer in HEK293 cells was not significantly higher than that of G4gl, and at the highest concentration of 100 µM, it was even lower. A completely different phenomenon was observed in HaCaT, HepG2, and U-118 MG cells, with the biotinylated G4Bgl dendrimer always accumulating in cells at a significantly higher level than its non-biotinylated analog [46]. This may indicate the possibility of an efflux of biotinylated neutral dendrimers from HEK293 cells or elimination of the advantage of biotinylated nanoparticle retention after a longer time or at higher concentrations over non-biotinylated ones. Weiner and Wolf showed high biotin retention over 24 h in rat hepatocytes [80]; however, Zempleni and Mock observed triphasic biotin efflux from peripheral blood mononuclear cells after 0.2, 1.2, and 22 h [81]. This phenomenon requires further, more detailed studies. Figure 9 show the relationships between the surface chemistry of G4, G4B, G4gl, and G4Bgl and cellular uptake, accumulation, and toxicity against HEK293 cells.

## 4. Materials and Methods

### 4.1. Materials

Fourth–generation polyamidoamine dendrimers (PAMAM G4) were synthesized and characterized as described earlier [46]. Biotin N-hydroxysuccinimide ester (biotin-NHS) and other standard chemicals were sourced from Merck (KGaA, Darmstadt, Germany). Sulfo-cyanin5 succinimidyl ester was obtained from MedChemExpress (Sollentuna, Sweden). Spectra/Por^®^ 3RC dialysis membranes (cellulose, MWcutoff = 3.5 kDa) were supplied by Carl Roth GmbH&Co KG (Karlsruhe, Germany). Eagle’s Minimum Essential Medium (EMEM) was acquired from ATCC (Manassas, VA, USA), and Dulbecco’s Modified Eagle’s Medium (DMEM) and fetal bovine serum (FBS) were provided by Corning Incorporated (Corning, NY, USA). Trypsin–EDTA solution was purchased from Capricorn Scientific (Ebsdorfergrund, Germany). Dulbecco’s phosphate-buffered saline (PBS) without magnesium and calcium ions and DAPI (4′,6-diamidino-2-phenylindole, dihydrochloride) were sourced from Thermo Fisher Scientific (Waltham, MA, USA). The phosphate-buffered saline (PBS) was provided by Roth (Karlsruhe, Germany). XTT sodium salt (2,3-bis [2-methoxy-4-nitro-5-sulfophenyl]-2Htetrazolium-5-carboxanilide inner salt) was obtained from Cayman (Ann Arbor, MI, USA). Phenazine methosulfate (PMS), N-methyl dibenzopyrazine methyl sulfate, penicillin and streptomycin solution, 0.4% trypan blue solution, and ATTO 590-Biotin BioReagent were purchased from Sigma–Aldrich (St Louis, MO, USA). All electrophoresis and Western blot reagents came from Bio-Rad (Hercules, CA, USA), and cell culture dishes were provided by Corning Incorporated (Corning, NY, USA) or Nunc (Rochester, NY, USA).

### 4.2. Cell Culture

Human embryonic kidney HEK293 cells were obtained from ATCC (Manassas, VA, USA) and cultured in EMEM supplemented with 10% heat-inactivated FBS, 100 U/mL penicillin, and 100 µg/mL streptomycin. The cells were maintained at 37 °C in a humidified environment with 5% CO_2_. Media were changed every 2–3 days, and the cells were passaged at 70–80% confluence after treatment with 0.25% trypsin–EDTA/PBS (calcium- and magnesium-free). Cell morphology was assessed using a Nikon TE2000S Inverted Microscope (Tokyo, Japan) with phase contrast, while cell counts and viability were determined via the trypan blue exclusion test using a TC20™ Automatic Cell Counter (Bio-Rad Laboratories, Hercules, CA, USA).

### 4.3. SMVT Expression

HEK293 cells were cultured to 70–80% confluence as described. Harvested cells were lysed using 50 µL of RIPA lysis buffer (Millipore, Burlington, MS, USA #20-188) per 1 × 10⁶ cells with protease inhibitors for 30 min at 4 °C, followed by centrifugation at 13,000 rpm for 15 min at 4 °C. For Western blotting, 15 µL of cell lysates (equivalent to 3 × 10⁵ cells) and 5 µL of Precision Plus Protein Dual Color Standards (Bio-Rad, Hercules, CA, USA #161-0374) were loaded onto a 5% stacking gel, and SDS-PAGE was performed on a 10% resolving gel. After separation, proteins were transferred onto Immun-Blot PVDF membranes (Bio-Rad, #162-0177) and blocked for two hours in 1% BSA in TBST (0.05% Tween 20, pH 7.5) at room temperature on a rocking platform. The sodium-dependent multivitamin transporter (SMVT) was detected at 68 kDa using a 1:500 dilution of an anti-SLC5A6 polyclonal antibody (Sigma–Aldrich, Saint Louis, MO, USA #SAB4503495) incubated in 1% BSA/TBST for two hours at room temperature, followed by a 1:5000 dilution of goat anti-rabbit IgG-HRP secondary antibody (Jackson ImmunoResearch, West Grove, PA, USA #111-035-003) for one hour. The bands were detected colorimetrically with DAB-buffer tablets (Millipore–Sigma, Burlington, MS, USA #EMD1.02924.0001) for 5 min.

### 4.4. Time- and Dose-Dependent Biotin Uptake

Cells were seeded in flat-bottom 96-well plates at a density of 4 × 10^4^ cells/well and incubated for 12 h. Biotin fluorescently labeled with ATTO 590 at the 0.01 or 0.1 µM concentration was prepared in DMEM culture media and added to HEK293 cells for 1, 3, or 6 h. Then, the medium was removed, the cells were washed with warm PBS, and the fluorescence intensity was measured with an Infinite M200 PRO (TECAN Group Ltd., Männedorf, Switzerland) at 580/635 nm (ATTO 590) against a blank sample (medium without cells).

### 4.5. Biotin and Derivatives’ Binding to the Monocarboxylate Transporter 1 (MCT-1)

The sequence of MCT-1 (UniProt ID: P53985) was used as a query for the Protein Data Base [82] at the RCSB PDB [82,83] portal (https://www.rcsb.org/ (accessed on 11 February 2025)). The obtained results were analyzed, and the template with the best possible sequence coverage and model quality was selected. Identification of the putative binding sites was performed in PyMOL (opensource ver. 3.0.0). Cavities were calculated using the CavitOmiX (v. 1.0, 2022, Innophore GmbH) PyMOL plugin. The corresponding hydrophobicity module of the program VASCo was used to analyze the hydrophobicity of the cavities [84], and the cavities were calculated using a modified LIGSITE algorithm [85]. Identified cavities representing putative binding sites were used as starting points in the performed docking simulations using tools provided by DockThor [86], a free web server for protein–ligand docking, (https://dockthor.lncc.br/ (accessed on 11 February 2025)) using a blind docking approach algorithm. The search box was centered at 117.485, 106.338, 100.240 Å for the structure representing an outward open conformation. The size of the box was kept at 40, 40, 40 Å. The remaining parameters were kept at default values. The ligands’ information was obtained from the PubChem database (https://pubchem.ncbi.nlm.nih.gov/ (accessed on 11 February 2025)), including biotin, biotin–NHS, and ATTO-labeled 590 biotin. For ATTO 590-labeled biotin, the ligand moiety was divided into two parts containing biotin and a fluorophore moiety before performing the docking experiments.

### 4.6. Cytotoxicity

Cells were seeded in flat-bottom, 96-well plates at a density of 1 × 10^4^ cells per well and allowed to adhere for 24 h. Working solutions of PAMAM G4 dendrimers and their conjugates (3.125–100 µM) in culture media were added to the cells. Following 48 h of exposure, the medium was removed, and an XTT mixture comprising 1.7 mM XTT and 8.3 µM PMS in complete medium was added at 100 µL per well. The plates were incubated at 37 °C for another 1 h. Absorbance was then measured at 450 and 620 nm against a blank sample (complete growth medium with XTT and PMS) as the reference with a microplate reader (µQuant–BioTek, Winooski, VT, USA). Results were reported as a percentage relative to that of the untreated control.

### 4.7. Time-Dependent Dendrimer Uptake

Cells were cultured as previously described. Solutions of Cy5-labeled dendrimers—G4, G4B, G4gl, and G4Bgl—at concentrations of 0.01 and 0.1 µM in EMEM were added to HEK293 cells for incubation periods of 1, 3, or 6 h (100 µL per well). Following incubation, the medium was removed, the cells were washed with warm PBS, and fluorescence intensity was measured at 640/680 nm (Cy5) using an Infinite M200 PRO plate reader (TECAN Group Ltd., Männedorf, Switzerland) with a blank sample (complete medium without cells) as a reference.

### 4.8. Proliferation and Dendrimer Accumulation

The impact of the tested compounds on cell proliferation was evaluated using DNA quantification estimated with DAPI staining. For this analysis, 4 × 10^3^ cells per well were seeded in flat-bottom, 96-well plates and incubated for 24 h at 37 °C to allow attachment. After removing the culture medium, the cells were treated with increasing concentrations (3.125–100 µM) of the tested dendrimer conjugates for 48 h. Following treatment, the medium was removed, and the cells were fixed in 3.7% formalin and then stained with a 600 nM DAPI solution in PBS for at least 1 h. Fluorescence was measured at 360/460 nm (DAPI) using an Infinite M200 PRO microplate reader (TECAN Group Ltd., Männedorf, Switzerland) against a blank sample (wells without cells), with the cell count directly correlating to fluorescence intensity.

Dendrimer accumulation after 48 h of incubation was measured by measurement of Cy5 fluorescence intensity with an Infinite M200 PRO microplate reader (TECAN Group Ltd., Männedorf, Switzerland) at 640/680 nm against a blank (wells without cells). Fluorescence intensity was converted to an equal number of cells by referencing the fluorescence intensity of Cy5 to the signal derived from DAPI. After measurement, images were collected with the fluorescence microscope Delta Optical IB-100 (Warsaw, Poland) with an excitation filter of 510–550 nm and an emission filter of 590 nm.

### 4.9. Statistical Analysis

To assess differences between treated and untreated control samples, a non-parametric Kruskal–Wallis test with significance set at *p* ≤ 0.05 was used, as the data did not follow a normal distribution (determined by the Shapiro–Wilk test). To compare biotinylated and non-biotinylated dendrimers’ accumulation at particular concentrations, the Mann–Whitney U test was performed. All statistical analyses and calculations were conducted using Statistica 13.3 software (StatSoft, Tulsa, OK, USA).

## 5. Conclusions

Human embryonic kidney HEK293 cells showed higher SMVT expression than human immortalized keratinocytes (HaCaT cells) and human glioma U-118 MG cells but lower expression than hepatocellular carcinoma HepG2 cells. At the same time, they took up fluorescently labeled biotin to the same extent as HaCaT cells, considered a model of normal cells, but to a lesser extent than glioma and liver cancer cells. The kinetics of the uptake of biotinylated dendrimers G4B and G4Bgl into HEK293 cells were similar to those observed for cells of other cell lines. Therefore, no relationship was detected between the level of SMVT expression and the efficiency of biotin and biotinylated cationic and neutral dendrimers’ uptake. Dendrimers’ accumulation was lower in HEK293 cells than in other cells, which may point to cellular efflux mechanisms. HEK293 cells showed high sensitivity to cationic G4 and G4B dendrimers and also low sensitivity to G4Bgl (similar sensitivity compared to U-118 MG cells but higher sensitivity than HepG2 and HaCaT cells). Therefore, they can be considered a good model of normal cells for studies on the use of cationic and neutral nanoparticles, such as biotinylated PAMAM dendrimers, in a manner rather independent of SMVT expression. The involvement of MCT-1 or other transporters in the cellular uptake of biotinylated PAMAMs should be taken into account.

## Data Availability

The original contributions presented in the study are included in the article; further inquiries can be directed to the corresponding author.
